# Effect of *Lactobacillus* spp. on adhesion, invasion, and translocation of *Campylobacter jejuni* in chicken and pig small-intestinal epithelial cell lines

**DOI:** 10.1186/s12917-020-2238-5

**Published:** 2020-02-03

**Authors:** Maja Šikić Pogačar, Tomaž Langerholc, Dušanka Mičetić-Turk, Sonja Smole Možina, Anja Klančnik

**Affiliations:** 1 Faculty of Medicine, University of Maribor, 2000 Maribor, Slovenia; 2 Department of Microbiology, Biochemistry, Molecular Biology and Biotechnology, Faculty of Agriculture and Life Science, University of Maribor, 2311 Hoče, Slovenia; 3 Department of Food Science and Technology, Biotechnical Faculty, University of Ljubljana, 1000 Ljubljana, Slovenia

**Keywords:** *Campylobacter jejuni*, *Lactobacillus* spp., Chicken and pig cell line, Adhesion, Invasion, Translocation

## Abstract

**Background:**

*Campylobacter* spp. are a major cause of bacterial food-borne diarrhoeal disease. This mainly arises through contamination of meat products during processing. For infection, *Campylobacter* spp. must adhere to epithelial cells of the mucus layer, survive conditions of the gastrointestinal tract, and colonise the intestine of the host. Addition of probiotic bacteria might promote competitive adhesion to epithelial cells, consequently reducing *Campylobacter jejuni* colonisation. Effect of *Lactobacillus* spp. (PCS20, PCS22, PCS25, LGG, PCK9) on *C. jejuni* adhesion, invasion and translocation in pig (PSI cl.1) and chicken (B1OXI) small-intestine cell lines, as well as pig enterocytes (CLAB) was investigated.

**Results:**

Overall, in competitive adhesion assays with PSI cl.1 and CLAB cell monolayers, the addition of *Lactobacillus* spp. reduced *C. jejuni* adherence to the cell surface, and negatively affected the *C. jejuni* invasion. Interestingly, *Lactobacillus* spp. significantly impaired *C. jejuni* adhesion in three-dimensional functional PSI cl.1 and B1OXI cell models. Also, *C. jejuni* did not translocate across PSI cl.1 and B1OXI cell monolayers when co-incubated with probiotics. Among selected probiotics, *Lactobacillus rhamnosus* LGG was the strain that reduced adhesion efficacy of *C. jejuni* most significantly under co-culture conditions.

**Conclusion:**

The addition of *Lactobacillus* spp. to feed additives in livestock nutrition might be an effective novel strategy that targets *Campylobacter* adhesion to epithelial cells, and thus prevents colonisation, reduces the transmission, and finally lowers the incidence of human campylobacteriosis.

## Background

*Campylobacter jejuni* is the most reported food-borne pathogen in human gastrointestinal infections over last decade [1]. *C. jejuni* are frequently found in the gastrointestinal tract of healthy animals that are destined for human consumption, especially chickens, which naturally harbour *Campylobacter* spp. in their gastrointestinal tracts [1–3].

The intestinal epithelium of the host represents the first barrier against this food-borne pathogen and is supported by the response of the mucosal immune system that is tightly connected with the gastrointestinal barrier. However, to establish an infection, *Campylobacter* spp. first adhere to and persist in the mucus layer that covers the intestinal epithelium, and thus survive the adverse conditions of the gastrointestinal tract, to finally colonise the jejunum and ileum of the host [4, 5]. Adhesion to epithelial cells of the animal gastrointestinal tract is thus the first and important step for successful colonisation. This further promotes transmission of *C. jejuni* to humans, which occurs mainly through handling and consumption of contaminated poultry and pork meat products during slaughter and carcass processing [2, 6, 7]. It is therefore imperative to reduce the prevalence and colonisation of *Campylobacter* at the farm level, where good hygiene and biosecurity practices are not sufficient [8]. In particular, the control of *Campylobacter* spp. in poultry is the most important concern for consumers [1, 9]. Importantly, this needs to be achieved without increased use of antibiotics and for that reason, alternative strategies for the reduction of colonisation of *Campylobacter* spp. are urgently needed. In this context, an effective approach that targets *Campylobacter* adhesion to the intestinal mucus will prevent colonisation and thus reduce the bacterial load of *Campylobacter* spp. in live animals. The consequent reduced transmission from animal carcasses to humans will thus lower the risk to consumers.

Several strategies with limited efficacy have been applied to reduce the burden of *Campylobacter* spp. in the intestine of pig and poultry including vaccination, passive immunisation, bacteriophage therapy, bacteriocin application, organic acids, and medium chain fatty acids [1, 9–11]. On the other hand, probiotic bacteria can have high affinity for adherence to the mucosal wall, where they promote the integrity of the healthy functioning of the gastrointestinal barrier by decreasing paracellular permeability through strengthening the tight junctions [12–14]. Thus, the selected probiotic strains could offer an alternative method to reduce *Campylobacter* spp. load in animal farming.

The protective role of the probiotic bacteria against pathogens mostly lies in their competition for adhesion sites and nutrients, and their production of antibacterial substances [15]. With the emergence of serious antibiotic resistance in livestock breeding, farmers are considering the use of probiotics as feed additives in livestock nutrition, as this might induce immune system function and confer health benefits to the host animal [16–18]. Higher production of volatile fatty acids in the large intestine contributes to good *Lactobacillus* sp. growth in the caecum of pigs, that enhance digestion, improve nutrient absorption in the intestine and consequently increase the feed efficiency [17, 19].

However, host–pathogen–probiotic interactions must be defined before the application of any alternative strategy for pathogen reduction at the farm level, as defined by the regulatory agencies in many countries, including the European Union [7]. To avoid unnecessary usage of animals in the research projects along with ethical considerations, many highly differentiated cell lines were developed with the aim to establish comparable functionality to their in vivo counterparts. In contrast to the more expensive animal trials, cell-line models are cost effective and allow massive screening. In addition, cell-line models are in line with the three R paradigms meaning to Reduce, Refine, and Replace [20]. Despite intense focus on probiotic research in literature, the *Campylobacter*-host cell-probiotic interaction mechanisms are not yet completely understood. *C. jejuni* is able to adhere to the gut epithelium, induce cell death, and disrupt mucosal barrier function. Although *Lactobacillus* spp. can modulate epithelial cell invasion by *C. jejuni*, how this is achieved is still not well understood [15, 21, 22]. A few studies have reported reduced *C. jejuni* adhesion when such probiotics had colonised the mucus, which has been studied in models with mucin [23] and with chicken intestinal mucus [24, 25]. However, the majority of in vitro models that have been used to investigate anti-*Campylobacter* activities of probiotics had been based on human cervical or intestinal cell lines [26]. The use of functional pig and chicken cell line models of non-tumorous origins may provide a better model if the goal is to identify probiotics that can be used in livestock nutrition. However, to the best of our knowledge, there have not been any in vitro studies that have evaluated the efficacy of *Lactobacillus* spp. for prevention of *C. jejuni* adhesion and invasion of pig and chicken intestinal epithelium.

Thus in our study we have used *Lactobacillus spp.* to modulate *Campylobacter* host-cell interactions using pig and chicken small-intestine cell-line models in vitro. The aim was to prevent and/or reduce the *C. jejuni* K49/4 adhesion, invasion and translocation through competitive adhesion with *L. plantarum* (PCS20, PCS22, PCS25), *L. rhamnosus* LGG and *L. plantarum* (PCK9), using pig (PSI cl.1) and chicken (B1OXI) epithelial small intestine cells, and pig enterocytes (CLAB cells).

## Results

### Cytotoxicity

**Fig. 1:**
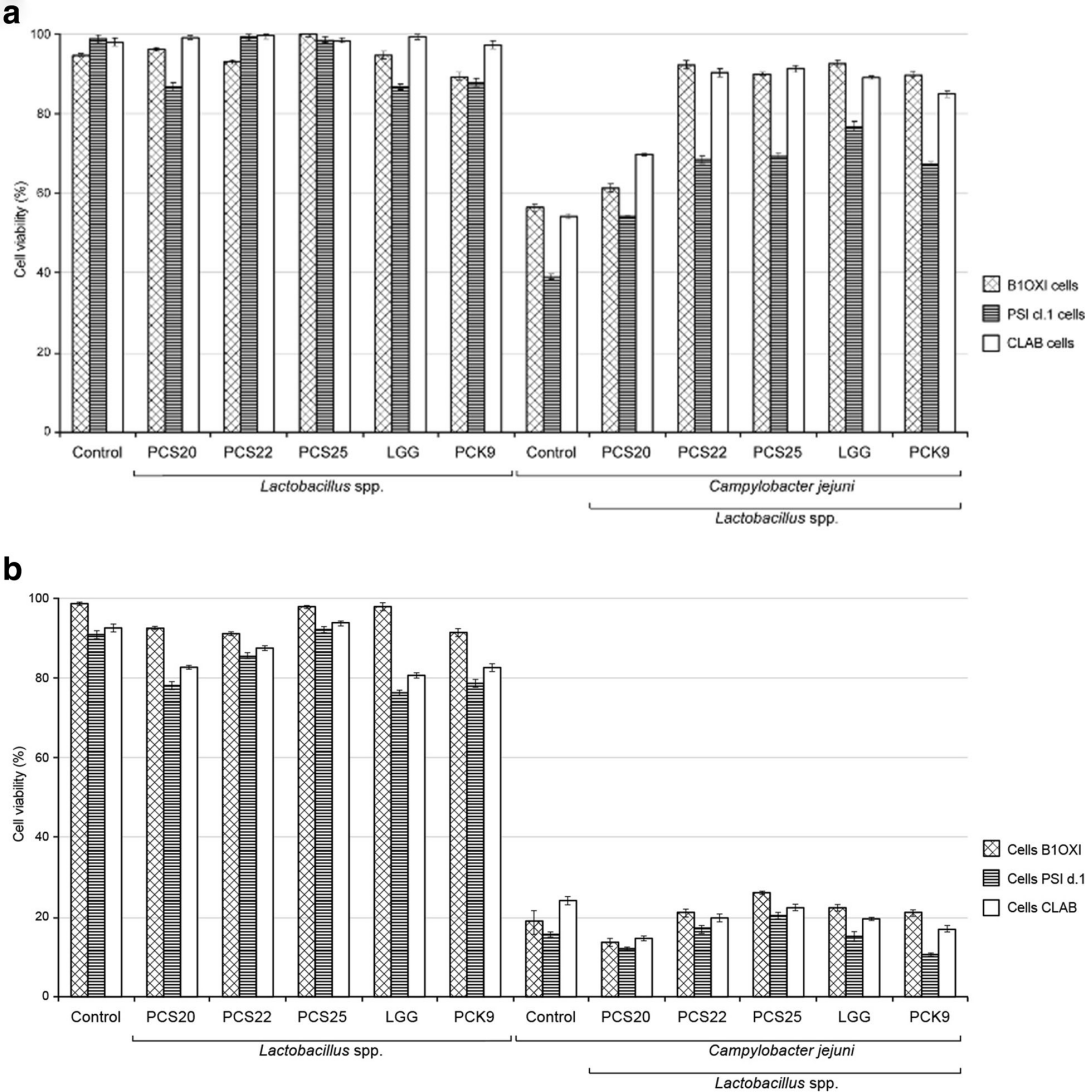
MTT proliferation assay. PSI c1.1, B1OXI and CLAB cells were seeded at 6 × 10^5^ cells/ well. As confluent monolayers, the cells were exposed to the selected probiotic bacteria (1 × 10^8^ CFU/mL) or to the combination of the probiotic bacteria (1 × 10^8^ CFU/mL) and *C. jejuni* (2 × 10^8^ CFU/mL), for 24 h (**a**) and 48 h (**b**). Error bars indicate the standard deviation from at least three independent experiments. Data are expressed as % cell survival, compared to the control

The co-incubation of the probiotic bacteria *L. plantarum* (PCS20, PCS22, PCS25), *L. rhamnosus* LGG and *L. plantarum* PCK9 (1 × 10^8^ CFU/mL) with the pig PSI cl.1, chicken B1OXI, and pig CLAB epithelial cell monolayers did not show any cytotoxic effects after 24 h. The viability of PSI cl.1, B1OXI and CLAB cells ranged from 90 to 100% when the probiotic bacteria were added, compared to non-treated monolayers (Fig. 1a). As expected, the co-incubation of pathogenic *C. jejuni* K49/4 with the PSI cl.1, B1OXI and CLAB cells resulted in cytotoxic effects, with disruption of the monolayers, when compared to untreated cells. This was most significant for the PSI cl.1 cells, which showed only 40% viability after 24 h (Fig. 1a), thus indicating that the PSI cl.1 cells were the most sensitive cells to *C. jejuni* infection. Addition of the probiotic strains in combination with *C. jejuni* showed protected effects for the PSI cl.1, B1OXI and CLAB cells viability. The viability of B1OXI and CLAB cells remained > 90% after the co-incubations of the combinations of the PCS22, PCS25, LGG and PCK9 bacteria with *C. jejuni* for 24 h (Fig. 1a), and more than 80% after 48 h (Fig. 1b). However, these combinations of the probiotic strains and *C. jejuni* showed greater detrimental effects with the PSI cl.1 cells, resulting in only 50 to 70% cell viability after 24 h of co-incubation (Fig. 1a). The viability of B1OXI, PSI cl1 and CLAB was even more reduced after 48 h of co-incubation, with less than 20% cell viability (Fig. 1b).

### *Campylobacter jejuni* adhesion and invasion in cell monolayers

**Fig. 2:**
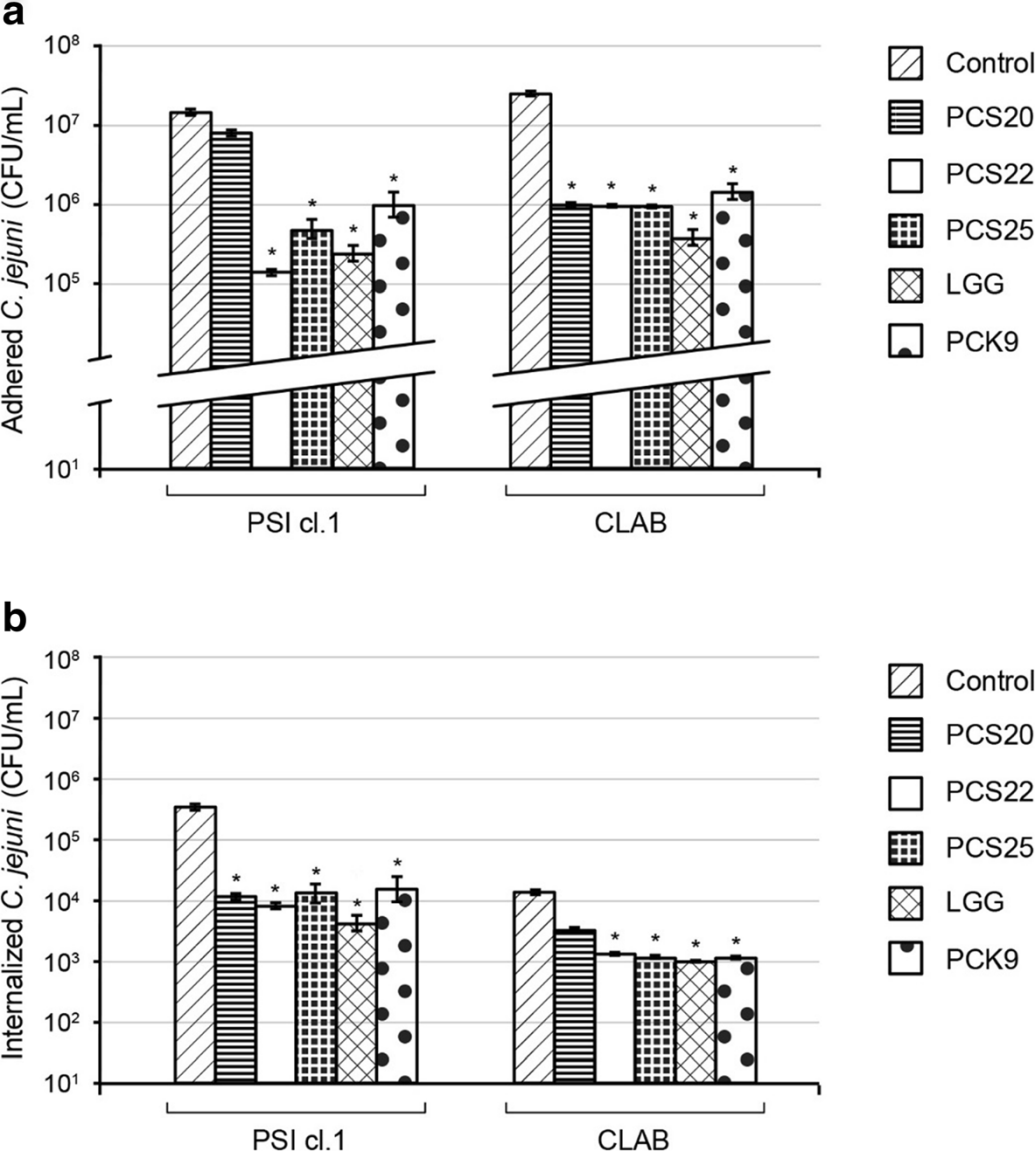
*Campylobacter jejuni* (2 × 10^8^ CFU/mL) adhesion to (**a**) and invasion into (**b**) non polarised PSI cl.1 and CLAB pig intestine epithelial cells, after 2 h co-culture without or with probiotic bacteria (as indicated; concentration of 1 × 10^8^ CFU/mL). Error bars indicate the standard deviation from at least three independent experiments. Data are expressed as means ±SD. *P ≤ 0.01, versus control

The efficiency of the *Lactobacillus* strains PCS20, PCS22, PCS25, LGG and PCK9 was determined in terms of impairment of competitive *C. jejuni* K49/4 adhesion and invasion to pig PSI cl.1 and CLAB enterocytes using the non-polarised cell models (Fig. 2). *L. rhamnosus* LGG showed the greatest reduction of *C. jejuni* adherence and invasion to the PSI cl.1 and CLAB cell monolayers. Here, the control adhesion of the starting *C. jejuni* inoculum was 2%, which was reduced approximately to 0.02% by *L. rhamnosus* LGG in both, PSI cl.1 and CLAB cell models (Fig. 2a). Additionally, *L. plantarum* PCS25 reduced this control *C. jejuni* adhesion on PSI cl.1 cells to 0.04% and PCS22 to only 0.01% (Fig. 2a). Despite slightly higher *C. jejuni* adhesion to CLAB cells (Fig. 2a), the *C. jejuni* invasion into these cells was lower when compared to the PSI cl.1 cells (Fig. 2b). As shown in Fig. 2b, < 0.1% of the *C. jejuni* cells invaded the PSI cl.1 cells, and approximately 0.001% invaded the CLAB cells. The results further demonstrate the efficiency of the *Lactobacillus* strains (PCS20, PCS22, PCS25, LGG, PCK9) to reduce *C. jejuni* K49/4 invasion into intestinal epithelial PSI cl.1 and CLAB cells as the monolayer model. The *C. jejuni* invasion rate into these cell monolayers was reduced by 90% when the probiotic bacteria where used (Fig. 2b). Thus, the competitive adhesion assays using PSI cl.1 and CLAB cell monolayers showed that addition of probiotic bacteria efficiently reduces the *C. jejuni* adherence to the surface of intestinal epithelial cells, and their invasion of these cells.

### *Campylobacter jejuni* adhesion and invasion using the polarised cell model

**Fig. 3:**
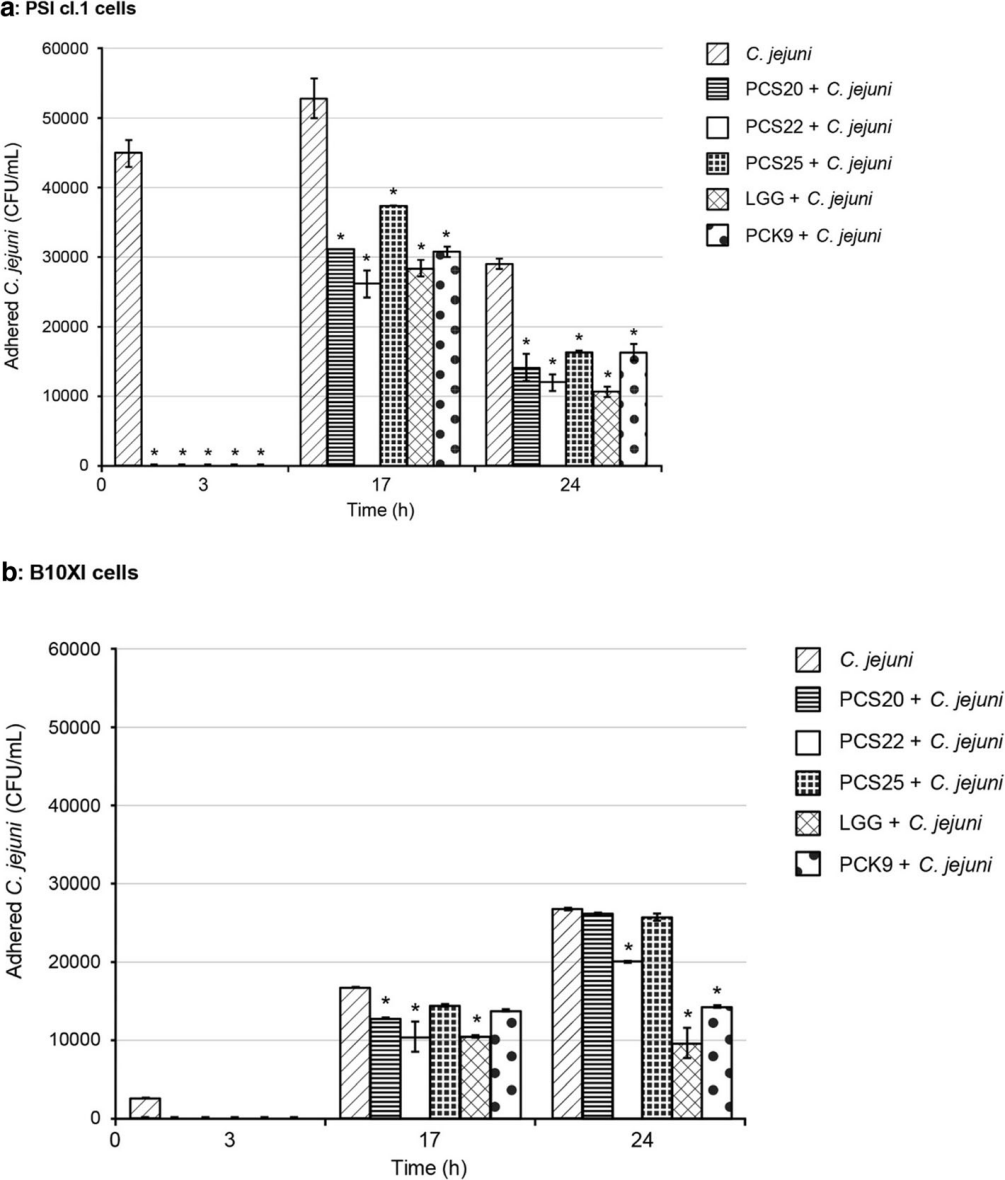
Effects of the probiotic bacteria on adhesion of *C. jejuni* using polarised pig PSI cl.1 (**a**) and chicken B1OXI (**b**) intestine epithelial cells, after 2 h co-culture without and with putative probiotic bacteria. Error bars indicate the standard deviation from at least three independent experiments. Data are expressed as means ±SD. *P ≤ 0.01, versus control

**Fig. 4:**
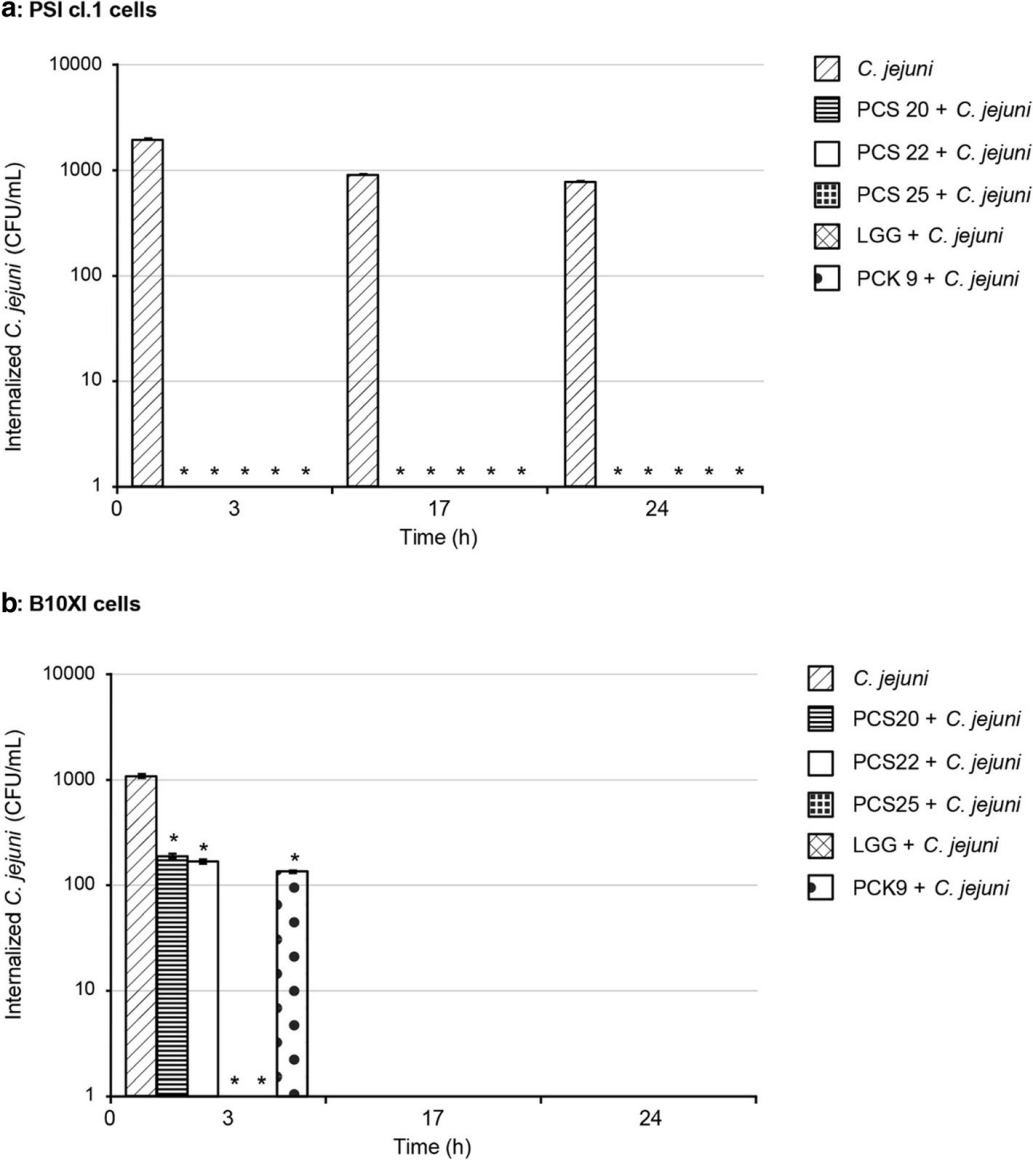
Effects of probiotic bacteria on internalization of *C. jejuni* using polarised pig PSI cl.1 (**a**) and chicken B1OXI (**b**) intestine epithelial cells, after 2 h co-culture without and with probiotic bacteria. Error bars indicate the standard deviation from at least three independent experiments. Data are expressed as means ± SD. *P ≤ 0.01, versus control

The efficiency of *Lactobacillus* strains PCS20, PCS22, PCS25, LGG and PCK9 to impair *C. jejuni* K49/4 adhesion and invasion were also tested using the three-dimensional functional model of the pig PSI cl.1 and chicken B1OXI polarised intestine epithelial cells. An additional file shows this in more detail (see the Additional file 1). The post-infection growth kinetics (i.e. 3, 17 and 24 h) were determined for *C. jejuni* adhesion (Fig. 3) and invasion (Fig. 4). *C. jejuni* adhesion to PSI cl.1 cells was more pronounced, with more adhered *C. jejuni* observed 17 h post-infection, which then dropped off (Fig. 3a). On the other hand, there was an increase in number of *C. jejuni* on the chicken B1OXI cells from the beginning of the infection, with the highest levels reached at 24 h post-infection (Fig. 3b). The addition of the probiotic bacteria PCS20, PCS22, PC25 and PCK9 significantly decreased *C. jejuni* adherence to PSI cl.1 and B1OXI cells early in the observation period (i.e., after 3 h), compared to non-treated cells (Fig. 3). Addition of PCS20, PCS22, PCS25 and PCK9 cells also significantly decreased the number of *C. jejuni* adhered to PSI cl.1 cells after 17 h and 24 h (Fig. 3a). However, for the numbers of *C. jejuni* adhered to B1OXI cells, these remained at the control levels 24 h post-infection when PCS20 and PCS25 were used, and only the PCS22, LGG and PCK9 significantly reduced these numbers (Fig. 3b). The control *C. jejuni* invasion into PSI cl.1 cells remained constant to 24 h, but was comparable to B1OXI cells only at 3 h post-infection (Fig. 4). Addition of PCS20, PCS22, PCS25 and PCK9 reduced the invasiveness of *C. jejuni* into PSI cl.1 cells to below the level of detection regardless of the time post-infection (Fig. 4a). *C. jejuni* invasion into B1OXI cells was only seen at 3 h post-infection, and addition of PCS20, PCS22 and PCK9 significantly reduced this *C. jejuni* invasiveness (Fig. 4b). Strains PCS25 and LGG were the two most efficient strains in preventing the invasion of *C. jejuni* into B1OXI cells when compared to other strains. When these two strains were co-incubated with *C. jejuni*, no invasion into B1OXI was observed 3 h post-infection (Fig. 4b).

### Intestinal epithelium integrity and *C. jejuni* translocation

The efficiency of *Lactobacillus* strains PCS20, PCS22, PCS25, LGG and PCK9 to impair the effects to *C. jejuni* K49/4 on epithelial intercellular integrity were investigated. These were determined by measurements of TEER immediately after infection of polarised PSI cl.1 and B1OXI cells, and after 3, 17 and 24 h post-infection.

**Fig. 5:**
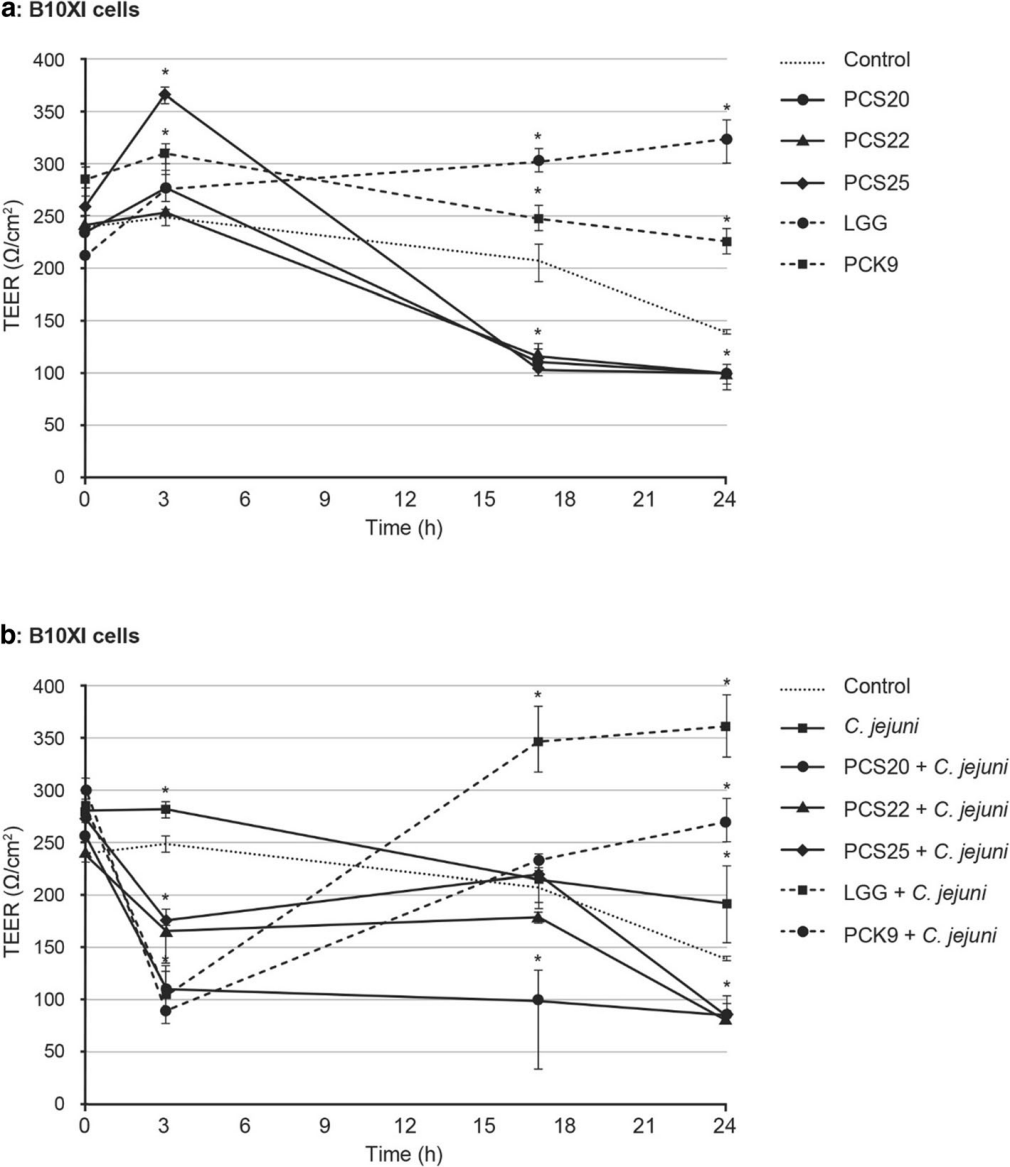
Transepithelial electrical resistance (TEER). PSIc1.1 (not shown) and B1OXI cells (**a** and **b**) were seeded at 6 × 10^5^ cells/well, as functional cell models using 12-well Transwell plates. As confluent monolayers, cells were exposed to the selected probiotic bacteria (1 × 10^8^ CFU/mL) and *C. jejuni* (2 × 10^8^ CFU/mL) for 2 h. Error bars indicate the standard deviation from at least three independent experiments. Data are expressed as means ±SD. *P ≤ 0.01, versus control

Initially, the effects of the probiotic bacteria on epithelial intercellular integrity of the PSI cl.1 and B1OXI cells were determined. At 1 × 10^7^ CFU/mL, the probiotic bacteria generally increased the TEER of the PSI cl.1 (data not shown) and B1OXI (Fig. 5a) polarised monolayers over the first 3 h of exposure (except for PCS22), as compared to the control (*p* < 0.01). By 24 h post-infection, the TEER in the presence of the probiotic bacteria PCS20, PCS22 and PCS25 decreased to below the control value. The exceptions of LGG and PCK9 maintained significantly increased TEER to 24 h post-infection with the B1OXI polarised monolayer, as compared to control (*p* < 0.01) (Fig. 5a).

The *C. jejuni* adherence to PSI cl.1 and B1OXI (Fig. 5b) polarised cells decreased the TEER to 24 h post-infection. For the B1OXI polarised cells, the addition of all of the probiotics to *C. jejuni* significantly decreased TEER in the first 3 h post-infection, a situation generally maintained to 24 h post-infection, for PCS20, PCS22 and PCS25. In contrast, addition of LGG and PCK9 resulted in increased TEER of B1OXI polarised cells beyond these first 3 h post-infection (Fig. 5b). Incubation of the PSI cl.1 polarised cells with *C. jejuni* caused additional decline in TEER values when compared to the probiotic bacteria only. Interestingly, no significant differences were observed for PSI cl.1 polarised cells between the probiotic strains (*p* > 0.01). The TEER of PSI cl.1 monolayers with no bacteria added was relatively constant over the 24 h of monitoring (≈1850 Ω/cm^2^).

**Fig. 6:**
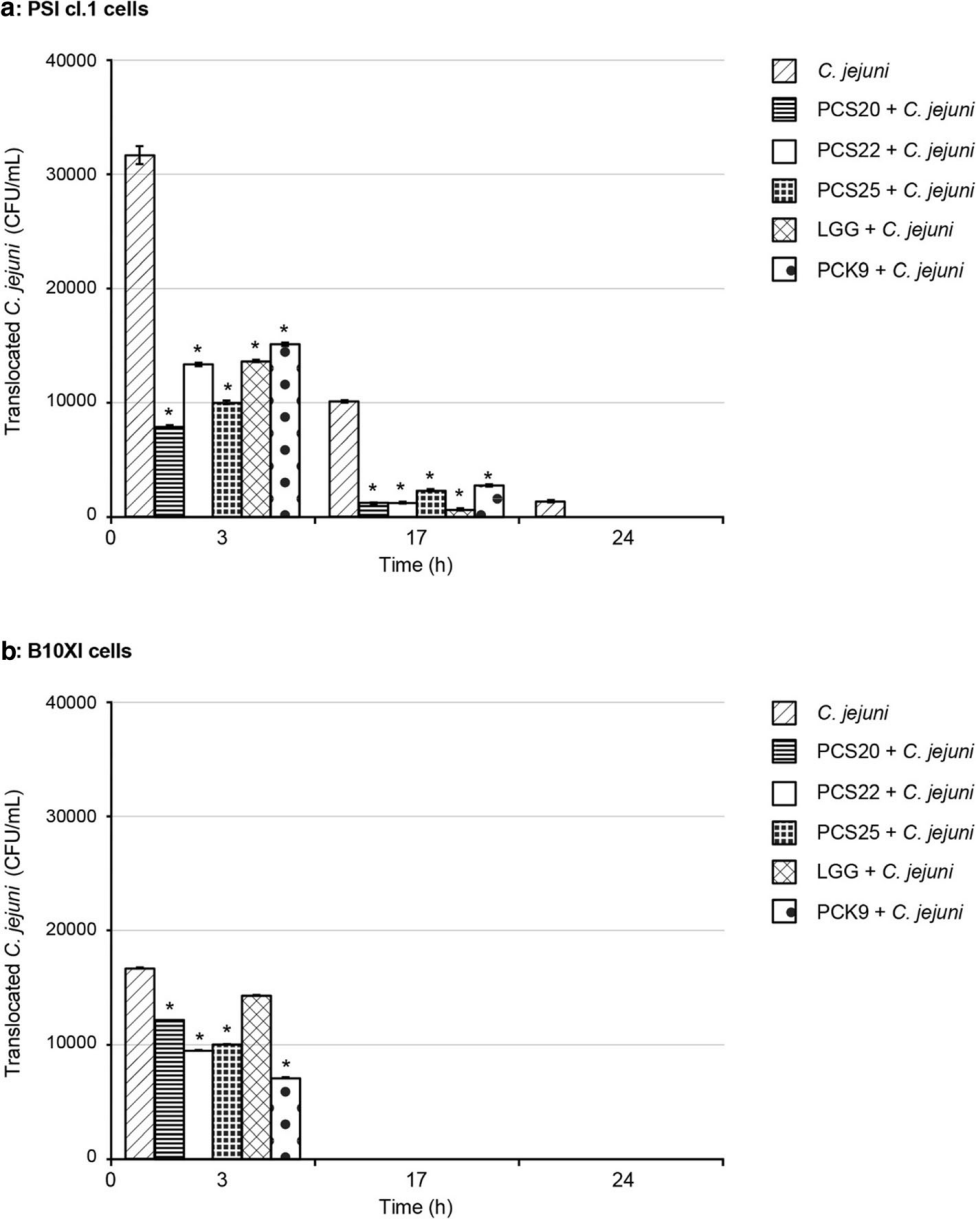
Translocation of *C. jejuni* K49/4 (2 × 10^8^ CFU/mL) through polarised PSI cl.1 (**a**) and B1OXI (**b**) intestine epithelial cells. Error bars indicate the standard deviation from at least three independent experiments. Data are expressed as means ±SD of bacteria detected in the basolateral chamber of the transwells. *P ≤ 0.01, versus control

*C. jejuni* K49/4 translocated to the basolateral compartment after apical infection of PSI cl.1 and B1OXI cells, as seen at 3 h post-infection (Fig. 6). Differences were seen when comparing *C. jejuni* translocation through these PSI cl.1 and B1OXI polarised monolayers. By 24 h, 1 × 10^2^ *C. jejuni* CFU/mL were present in the basolateral compartment for PSI cl.1 cells (Fig. 6a). No translocated bacteria were detected after 17 h post-infection for B1OXI cells (Fig. 6b). At 3 h post-infection, addition of PCS20, PCS22, PCS25, LGG and PCK9 significantly impaired *C. jejuni* translocation to the basolateral chamber of PSI cl.1 and B1OXI cells (Fig. 6). Of note, although *C. jejuni* adhered in high numbers to PSI cl.1 cells to 24 h (Fig. 3), the probiotic bacteria prevented *C. jejuni* invasion into these cells (Fig. 4), and consequently the *C. jejuni* translocation to basolateral chamber. These monolayers were not disrupted over the first 17 h post-infection, as was observed under the microscope. In addition, the TEER stayed relatively constant over this period, which indicated that the *C. jejuni* translocation was mostly transcellular.

## Discussion

Adhesion represents a virulence factor in human/animal infections that is important for *Campylobacter* survival out of the host and during host–pathogen interactions. *Campylobacter* adhesion to epithelial cells involves contact between the bacterial and the eukaryotic cell surfaces. It is also crucial for further *Campylobacter* invasion of the intracellular space and traversing of the host barriers, and thus for the *Campylobacter* pathogenicity [3, 27]. *Campylobacter* spp. of different origins can adhere to and invade human, porcine and avian cell lines [27–32]. The data in the present study on *C. jejuni* adhesion to and invasion of PSI cl.1, B1OXI and CLAB cell lines are in agreement with this. Furthermore, when non-polarised PSI cl.1 and CLAB cells were used, greater *C. jejuni* adhesion was seen for CLAB cells, which reflects the differences in the structural and functional characteristics of the cell surface. However, when intestinal cell lines are cultivated on microporous membranes such as in transwells, they can spontaneously differentiate and polarise. In this way they serve as a much better model of the in vivo intestinal environment. Another advantage of transwells is that they can be used for studies of transport through the polarised epithelial monolayer. In the present study, we examined the ability of selected probiotic strains to prevent and/or reduce *C. jejuni* K49/4 adhesion, invasion and translocation using different intestinal epithelial cell lines of non tumor origin, in order to stimulate more closely the host-pathogen-probiotic interactions. The cell lines were co-incubated with *C. jejuni* K49/4 and various probiotic strains in order to simulate a possible scenario of infection.

First, we demonstrated no cytotoxic activities of selected probiotic bacteria towards pig PSI cl.1, chicken B1OXI epithelial small intestine cells, and pig CLAB enterocytes. Our results showed that the most sensitive cell line to addition of *C. jejuni* was the PSI cl.1, while the B1OXI cells tolerated exposure to prolonged probiotic–pathogen interactions reflecting the pathological or commensal behaviour of *C. jejuni* in different intestinal epithelial cell line. Results on adhesion capacity showed that at 3 h post-infection, *C. jejuni* adhesion to polarised PSI cl.1 cells was greater in comparison to that with polarised B1OXI cells, which is in agreement with earlier studies [33, 34]. The numbers of *C. jejuni* that adhered to the polarised PSI cl.1 cells decreased towards the end of observation period (i.e., 24 h post-infection). As suggested by Van Deun et al. (2008) this might be a colonisation strategy of *Campylobacter* spp. involving rapid replication in the intestine mucus followed by invasion of the intestinal epithelium in order to avoid mucosal clearance [35]. In contrast, the numbers of adhered *C. jejuni* increased from the beginning of the infection on B1OXI cells, which demonstrates the host-specific tropism of this pathogen. This is in agreement with other studies that have demonstrated that within an avian host, *Campylobacter* behave as commensals and do not proliferate intracellularly [35, 36]. However, the differences in *C. jejuni* adhesion indicate strain-specific responses, and also different mechanisms of adhesion for these different cell lines, which is in agreement with other studies [24, 36, 37].

Furthermore, we aimed to prevent/reduce this adhesion to the non-polarised PSI cl.1 and CLAB cells and the polarised PSI cl.1 and B1OXI intestinal epithelial cells by addition of probiotics, and thus to potentially control *Campylobacter* contamination and/or transmission.

With the main emphasis on human health, this can be particularly effective in the first segment of the food chain, which is an animal breeding for meat production. This has become urgent because of the high *Campylobacter* prevalence, increased antibiotics usage and consequent bacterial resistance on livestock farms, and especially in broiler production [38]. Thus, one of the options is to reduce the prevalence of broiler flocks colonised with *Campylobacter*, and to reduce the intestinal *Campylobacter* load of the broiler chickens prior to slaughter [1, 8].

To avoid the selection pressure for the emergence of resistant bacteria and to avoid deleterious effects that antibiotics can have on the protective microbiota, research is ongoing into alternative strategies, including phage therapy and vaccination; however, none of these approaches have proven effective in practice to date [7, 39]. In our research, we have studied the use of probiotics as an alternative strategy. Probiotics are known to increase blood glucose and albumin contents which indicate a better digestion and absorption of nutrients in the broiler, they decrease blood triglycerides and cholesterol, promote the integrity of the healthy gastrointestinal barrier, and also to enhance the gut microbiota. They contribute to the general well being of the host due to symbiosis they establish within the host [18]. This might allow the prevention, and potentially the treatment, of a variety of human diseases, through delaying *C. jejuni* colonisation and the signal transduction that arises during *C. jejuni* infection [40, 41].

As observed, *Lactobacillus* spp. reduced *C. jejuni* adhesion to pig PSI cl.1 epithelial small intestine cells and CLAB enterocytes using a non-polarised cell model, with the greatest effects seen for *L. rhamnosus* LGG. We confirmed that the presence of these probiotic bacteria hindered the adherence of *C. jejuni* to the cell surface. We thus support the conclusion of Mohan (2015), who reported that particular probiotic strains can out-compete *C. jejuni* through different mechanisms of actions. Mohan (2015) particularly noted that the probiotics reached the adhesion sites more rapidly, with competitive exclusion against *C. jejuni* through occupation of the adhesion sites [42]. The present data (e.g. Figs. 2 and 3) show that *L. rhamnosus* LGG was the most effective among selected probiotics and reduced the most the adherence of *C. jejuni* to both polarised PSI cl.1 and B1OXI cells. This would appear to be mediated via the adherence of *L. rhamnosus* LGG to these intestine cells and its persistence there for prolonged periods after administration, as was also indicated by the study of Alander et al. (1999) [43]. In addition, while the numbers of adhered *C. jejuni* dropped towards the end of the observational period (24 h post –infection) in PSI cl 1 cells, the opposite was observed in B1OXI cells. This could reflect not only the different nature of interactions as pathogen or commensal in different host but also the different attachment capacity and affinity of probiotic strains for attachment sites. Once adhered to the host cell, *C. jejuni* can then invade the cell, and hence survive for long periods inside both phagocytes and epithelial cells [44, 45]. This is a further important factor in disease pathogenesis, and it is mainly due to cell death and/or disruption of the mucosal barrier function, and correlates with both pathogen virulence and disease severity [19].

When the probiotic bacteria were added in the present study, *C. jejuni* invaded the non-polarised PSI cl.1 cells in greater numbers than for the CLAB cells, which suggests that these probiotic strains were more efficient in preventing the invasion of the CLAB cells. According to other studies and knowing that *Campylobacter* spp. are commensals in poultry, we hypothesised that *C. jejuni* would not invade the chicken B1OXI epithelial intestinal cells in vitro [33, 34]. The data here show that *C. jejuni* invaded polarised PSI cl.1 cells in higher numbers than for the B1OXI cells, and also persisted in the PSI cl.1 cells through the whole observation period; instead, there were no *C. jejuni* in the B1OXI cells 17 h post-infection. The host immune response greatly affects *C. jejuni* colonisation, especially in the very early phase of colonisation [35]. Taking into account that adhered *C. jejuni* persisted attached to B1OXI cells for 24 h while not invading intestinal epithelium implies it is not recognized as pathogenic bacterium by chicken epithelial cells and continued to replicate attached to the surface of the cells.

The extent of inhibition of *C. jejuni* invasion was dependent on the probiotic strain. Each probiotic strain prevented the invasion of PSI cl.1 cells; however, only *L. plantarum* PCS25 and *L. rhamnosus* LGG completely inhibited *C. jejuni* invasion of B1OXI cells. Other in vitro studies have shown that the extent of reduction in adhesion and/or invasion rates due to probiotic use is highly strain specific [19, 21, 40, 42]. Campana et al. (2012) observed inhibitory activity of *L. acidophilus* ATCC4356 on *C. jejuni* adhesion to and invasion of Caco-2 cells [46]. More recently, Wang et al. (2014) isolated four adhesive *Lactobacillus* strains that inhibited *C. jejuni* adhesion to and invasion of human HT29 cells [47]. The presented data show that the invasion of intestinal cells in vitro can be completely prevented by the use of particular probiotic bacteria. Moreover, in agreement with other studies, the inhibition of *C. jejuni* invasion of intestinal epithelial cells might be attributed to competitive exclusion by the probiotic bacteria [48]. However, other mechanisms of action of probiotic bacteria in addition to competitive exclusion should be taken into account. This is particularly evident in the present study because better *C. jejuni* adhesion did not necessarily result in better invasion rates, and vice versa. In addition to different adherence properties of the probiotic bacteria to the intestinal epithelial cells in vitro, the production of various metabolites (e.g., bacteriocins) or alterations in the signalling cascades involved in the invasion should also be taken into account when interpreting these data, as these aspects are also likely to be modified by the probiotic bacteria.

Finally, the probiotic strains were tested for their efficacies for the prevention of *C. jejuni* translocation using the three-dimensional functional model of the pig (PSI cl.1) and chicken (B1OXI) polarised intestine epithelial cells. It would be reasonable to assume that prevention of *C. jejuni* adhesion to eukaryotic cells would prevent the translocation of this pathogen. Alemka et al. (2010) showed that probiotics attenuated *C. jejuni* adhesion to, invasion of, and translocation across polarised HT29-MTXE12 cells, which is a sub clone of the human colon carcinoma HT29 cell line that secretes mucus [36]. Contrary to our expectation, at 3 h post-infection, *C. jejuni* efficiently translocated across the polarised chicken B1OXI intestinal cells; interestingly, the translocation level was much lower when compared to PSI cl.1 cells. Similar observations were reported by Konkel et al. (1992), who initially showed the highest rate of *C. jejuni* translocation at 4 h post-infection for polarised Caco-2 cells [45]. When probiotics in the present study were incubated with *C. jejuni*, the levels of translocated *C. jejuni* were similar in both PSI cl.1 and B1OXI cell lines (Fig. 6). Later, despite high levels of adhered *C. jejuni* (Fig. 3b), no *Campylobacter* spp. were detected in the basolateral chamber of the B1OXI cells (Fig. 6b) and no monolayer disruption was observed under the microscope which suggested that no translocation occurred and that *C. jejuni* did not proliferate in the basolateral chamber. The route of *C. jejuni* translocation to the basolateral chamber of transwells has been the subject of a lot of research, and some studies have described *C. jejuni* transcellular transcytosis [49], while others have implicated a paracellular route of translocation [36, 50]. Changes in TEER of the infected PSI c1.1 cells during the first 24 h post-infection, and the persistence of *C. jejuni* in the basolateral chamber of the transwells, indicated here that *C. jejuni* used the transcellular rather than a paracellular route of translocation. There were also no correlations determined between the variations in the TEER of the cell lines and the translocation of *C. jejuni* across the polarised intestinal epithelial cell monolayers. Furthermore, an increase in TEER was observed for the polarised intestinal epithelial cell monolayers when the probiotic bacteria were co-incubated with *C. jejuni* indicating that latter contributed to the integrity of the intestinal epithelium.

## Conclusions

Contrary to the acute enterocolitis caused by *C. jejuni* in humans, poultry exposed to *Campylobacter* show prolonged colonisation without pathological changes in the intestine or clinical signs of infection. Furthermore, despite several control measures, animals (and especially chickens) remain the most common source of *Campylobacter* spp. Addition of *Lactobacillus* spp. to animal feed might represent an effective novel strategy to reduce the prevalence of *Campylobacter* spp. in broiler chickens and colonisation in animals prior to slaughter.

To the best of our knowledge, the present study is the first that uses functional pig and chicken cell line models of non-tumorous origins to study the effects of probiotics on *Campylobacter* adhesion, invasion and translocation*. Lactobacillus spp.* reduced *C. jejuni* adhesion to PSI cl.1 pig epithelial small intestine cells and CLAB pig enterocytes as cell monolayers, and negatively affected *C. jejuni* invasion into these cells. Additionally, the probiotics impaired *C. jejuni* adhesion, invasion and translocation across three-dimensional functional PSI cl.1 and B1OXI polarised intestine epithelial cell models. When probiotics in the present study were incubated with *C. jejuni*, the levels of translocated *C. jejuni* were comparable in both used cell lines. During the first 3 h post-infection, *C. jejuni* more efficiently translocated across the polarised PSI cl.1 cells. Afterwards, *C. jejuni* did not translocate across PSI cl.1 and B1OXI cell monolayers when co-incubated with probiotics. Among selected probiotics, *L. rhamnosus* LGG was the strain that reduced adhesion efficacy of *C. jejuni* most significantly under co-culture conditions. We thus propose that the addition of *Lactobacillus* spp. to animal feed would represent an effective novel strategy to target *Campylobacter* adhesion to epithelial cells, prevent *Campylobacter* colonisation, reduce *Campylobacter* transmission, and finally, minimize the risk of bacterial spread to humans. Further research is necessary to clarify the mechanism(s) of these probiotic–*Campylobacter* interactions and to investigate specific/ effective probiotic strains for attenuation of the virulence properties and to combat *C. jejuni* infections.

## Methods

### Bacterial strains and growth conditions

*Lactobacillus* strains used in this study were *Lactobacillus plantarum* PCS20, PCS22, PCS25 (cheese isolates, from the collection of the Department of Biochemistry and Nutrition, Faculty of Medicine, University of Maribor, Slovenia), *Lactobacillus rhamnosus* LGG (from American Type Culture Collection; ATCC53103) and *Lactobacillus plantarum* PCK9 (from a cheese isolate, obtained during the European research project PathogenCombat; FP6–007081) [51]. The strains applied to the intestinal epithelial cell lines in our study were selected on the basis of previous in vitro and in vivo research. All *Lactobacillus* strains were previously determined for their probiotic characteristics and showed good adhesive properties to intestinal epithelial cells [52–55]. The tested strains were grown in De Man Rogosa, Sharpe (MRS) broth (Merck, Darmstadt, Germany) for 24 h at 37 °C, and under anaerobic conditions. The final bacterial suspensions for the competition assays contained approximately 1 × 10^8^ CFU/mL.

*C. jejuni* K49/4, a poultry meat isolate was grown at 42 °C microaerophilically (5% O_2_, 10% CO_2_, 85% N_2_) on Columbia agar (Oxoid, Hampshire, UK) supplemented with 5% defibrinated horse blood (Oxoid, Hampshire, UK). *C. jejuni* were transferred to Preston broth (Oxoid) at 42 °C, and grown microaerophilically for 9 h. These *C. jejuni* cultures in exponential phase were diluted in cell culture media containing no antibiotics to approximately 2 × 10^8^ CFU/mL and were used for cell culture assays.

### PSI cl.1, B1OXI and CLAB cell monolayers

The normal PSI cl.1 epithelial-derived cell line (partially differentiated cryptic enterocyte-like) was obtained from an adult pig at slaughter, as previously described [20]. These cells represent the closest model to humans in terms of genome, organ development, anatomy, physiology and metabolism of the intestinal tract, and for disease progression, and intestine–microbe interactions [56, 57]. The B1OXI cells represent normal enterocytes of the chicken small intestine. The B1OXI cell line was developed and characterised at the University of Maribor, Slovenia, in the framework of the EU funded ‘PathogenCombat’ project, to build a three-dimensional functional epithelial cell model of the chicken intestine [51]. The CLAB cells are enterocytes that were obtained from an adult pig at slaughter in Slovenia and represent adult mucin secreting enterocyte-like cell line [56]. Although CLAB cells are epithelial in origin, they do not polarise in vitro [51]. These cells are of non-tumorigenic origin, which makes them more suitable as the in vitro model to study pathogen–host interactions than tumorigenic cell lines. The phenotypical and functional characterization of the cells was performed with immunocytochemistry and the search for the presence of key epithelial markers. In addition, all cell lines were tested for mycoplasma contamination prior to the experiments.

The PSI cl.1 and B1OXI cell lines can form a tightly packed epithelial barrier when grown on microporous inserts, and for this reason, they were chosen for further studies to evaluate the efficacy of the probiotics for prevention of *C. jejuni* K49/4 adhesion, invasion and translocation across polarised cell monolayers. PSI cl.1, B1OXI and CLAB cells are of non-tumorigenic origin; instead, they were isolated from dissected animal tissue using the limiting dilution technique. The functional PSI cl.1 and B1OXI intestinal cell models were developed for studies into probiotic/pathogen/gut epithelial interactions in more detail following initial screening for the efficacy of the probiotics for prevention of *C. jejuni* K49/4 adhesion and invasion on PSI cl.1 and CLAB cell monolayers.

### Cultivation and propagation of cell lines

PSI cl.1, B1OXI and CLAB cell lines were grown in advanced Dulbecco’s modified Eagle’s medium (DMEM) (Sigma-Aldrich, Grand Island, USA), supplemented with 5% foetal calf serum (Lonza, Basel, Switzerland), 2 mmol/L L-glutamine (Sigma), 100 U/mL penicillin (Sigma) and 1 mg/mL streptomycin (Fluka, Buchs, Switzerland), at 37 °C in a humidified atmosphere of 5% CO_2_. To form monolayers, 96-well microplates were seeded with approximately 5.0 × 10^5^ cells/mL PSI cl.1 and CLAB, and incubated for 24 h to reach confluence. Thereafter, the cultures were washed three times with phosphate-buffered saline (PBS) and cultivated in antibiotic free DMEM for the cell adhesion and invasion assays.

### PSI cl.1 and B1OXI polarised cell model

To obtain polarised monolayers, PSI cl.1 or B1OXI cells were seeded on Transwell filter inserts (pore size, 0.4 μm; 12 mm; Corning) that were placed into 12-well plates (22.1 mm, Corning), at a density of 1 × 10^5^ cells/cm^2^. The TEER was measured using an electrical resistance system (Millicell-ERS; Millipore, Bedford, MA, USA). The net TEER was corrected for background resistance by subtraction of the resistance of the microporous membranes with the cell cultures (108 Ω/cm^2^) from the resistances measured with the system. Functional polarity was established when the TEER between the apical and basolateral surfaces of the monolayers exceeded 1600 Ω/cm^2^ for PSI cl.1 cells and 240 Ω/cm^2^ for B1OXI cells. TEER was measured before the cells reached confluence, after the addition of the bacteria to the medium (with or without gentamicin), and 24 h post-infection. The TEER of cell monolayers without bacteria was used as the control for each experiment.

### Effects of probiotic bacteria and *C. jejuni* K49/4 on viability of cell cultures

To test the effects of the bacteria on the viability of the PSI cl.1, B1OXI and CLAB cell monolayers, the cells were seeded separately in microplates at a density of 6 × 10^5^ cells/well. Later, each strain of viable probiotic bacteria (1 × 10^8^ CFU/mL) and *C. jejuni* K49/4 (2 × 10^8^ CFU/mL) were added to pre-washed monolayers of the PSI cl.1, B1OXI and CLAB cells, and the cell monolayers were incubated for 24 h at 37 °C in a humidified atmosphere of 5% CO_2_. The 3-(4,5-dimethylthiazol-2-yl)-2,5-diphenyltetrazolium bromide (MTT) colorimetric assay was used to determine cell viability, as described previously [58]. For each probiotic strain and for co-incubation of *C. jejuni* with probiotic bacteria three wells were used for each replicate. The mean absorbance of the control wells containing only confluent cell culture without any bacteria was taken as 100%. The percentage of metabolically active cells treated with probiotic bacteria and *Campylobacter jejuni* was then calculated.

### Cell culture assays

#### Adhesion, invasion, and intracellular survival of C. jejuni K49/4 with PSI cl.1 and CLAB cells

Bacterial adhesion tests on PSI cl.1 and CLAB cell monolayers were carried out in 96-well tissue culture plates. After washing the cell monolayers with PBS, each of the probiotic bacteria (approximately 1 × 10^8^ CFU/mL) were added simultaneously with *C. jejuni* K49/4 (2 × 10^8^ CFU/mL) to each well. The control wells were prepared by adding *C. jejuni* K49/4 to the cell monolayers. The cells were incubated at 37 °C in 5% CO_2_ for 2 h, to allow adhesion and invasion. After washing with DMEM, DMEM containing 100 μg/mL gentamicin was added, to determine the number of invaded *C. jejuni.* After a 1-h incubation, the monolayers were lysed with 1 mL/L (v/v) Triton X-100, for 5 min and were serially diluted. The intracellular bacteria were determined by plate counting at 3, 9, 24 and 48 h post-infection. The total numbers of adherent and internalised bacteria were determined simultaneously by performing the invasion assay, but without gentamicin treatment. The differences between the numbers of total and intracellular bacteria were calculated as the number of adherent *C. jejuni* cells.

#### Adhesion, invasion, and translocation of C. jejuni K49/4 using the PSI cl.1 and B1OXI polarised cell model

A functional cell model using PSI cl.1 and B1OXI cells was developed to determine inhibitory effect of the probiotic bacteria on the *C. jejuni* K49/4 adhesion, invasion and translocation of *C. jejuni* to the basolateral compartment of the well. When cells were confirmed to have reached confluence using TEER (> 1600 Ω/cm^2^ for PSI cl.1 cells; > 240 Ω/cm^2^ for B1OXI cells), the monolayers were washed twice with 100 μL DMEM without antibiotic/supplements, and infection assays were performed by seeding the bacterial inoculum of each probiotic culture (approximately 1 × 10^8^ CFU/mL) together with *C. jejuni* K49/4 (approximately 2 × 10^8^ CFU/mL) in the apical chamber. The total numbers of adhered and invaded *C. jejuni* K49/4 were determined at 3, 17 and 24 h post-infection after lysing the cells by addition of 500 μL Triton X-100 and plating on Columbia agar plates. Bacterial count for infection of cell lines and chosen time intervals were similar as in our previous studies [32, 33]. In addition, the numbers of translocated *C. jejuni* K49/4 were determined at the same time intervals. To investigate the effects of *C. jejuni* K49/4 on PSI cl.1 and B1OXI cell monolayer integrity following the infection of the cell lines, the TEER was measured at 0, 3, 17, and 24 h post-infection. The TEER of the infected cells was compared to non-infected cells. Furthermore, the effects of co-incubation of probiotics with *C. jejuni* K49/4 were also assessed.

### Statistical analysis

To define the effects of lactobacilli on *C. jejuni* K49/4 adhesion, data from triplicate samples from at least three independent experiments were analysed statistically with the Predictive Analytics (PASW) statistics 202 software, version 18.0 (IBM Corp., Armonk, NY, USA), for the significance of any changes in bacterial numbers. Statistical analyses were performed with unpaired Student’s t-tests to estimate the statistical significance. All data are presented as means ±standard deviations (error bars) of the replicate experiments. All statistical values were considered significant at *P* ≤ 0.01.

## Supplementary information

**Additional file 1. **Visual scheme of experiment. We assessed the effect of different *Lactobacillus* strains on *C. jejuni* adhesion, invasion and translocation using pig (PSI) and chicken (B1OXI) enterocyte cell lines of non tumorigenic origin.

## Data Availability

The data that support the findings of this study are available from the corresponding author upon reasonable request.

## Abbreviations

B1OXI: chicken small-intestine cell line
*C. jejuni*: *Campylobacter jejuni*
DMEM: Dulbecco’s modified Eagle’s medium
MTT: 3-(4,5-dimethylthiazol-2-yl)-2,5-diphenyltetrazolium bromide colorimetric assay
PBS: phosphate-buffered saline
PCS20, PCS22, PCS25, LGG and PCK9: *Lactobacillus* strains
PSI cl.1 and CLAB: pig enterocytes
TEER: transepithelial resistance
