# Supplementary figures and images for: Effect of Lactobacillus spp. on adhesion, invasion, and translocation of Campylobacter jejuni in chicken and pig small-intestinal epithelial cell lines
Source: BMC Vet Res. 2020 Feb 3;16:34. doi: 10.1186/s12917-020-2238-5 (PMC6998324; doi:10.1186/s12917-020-2238-5)

**VISUAL SCHEME OF EXPERIMENT**


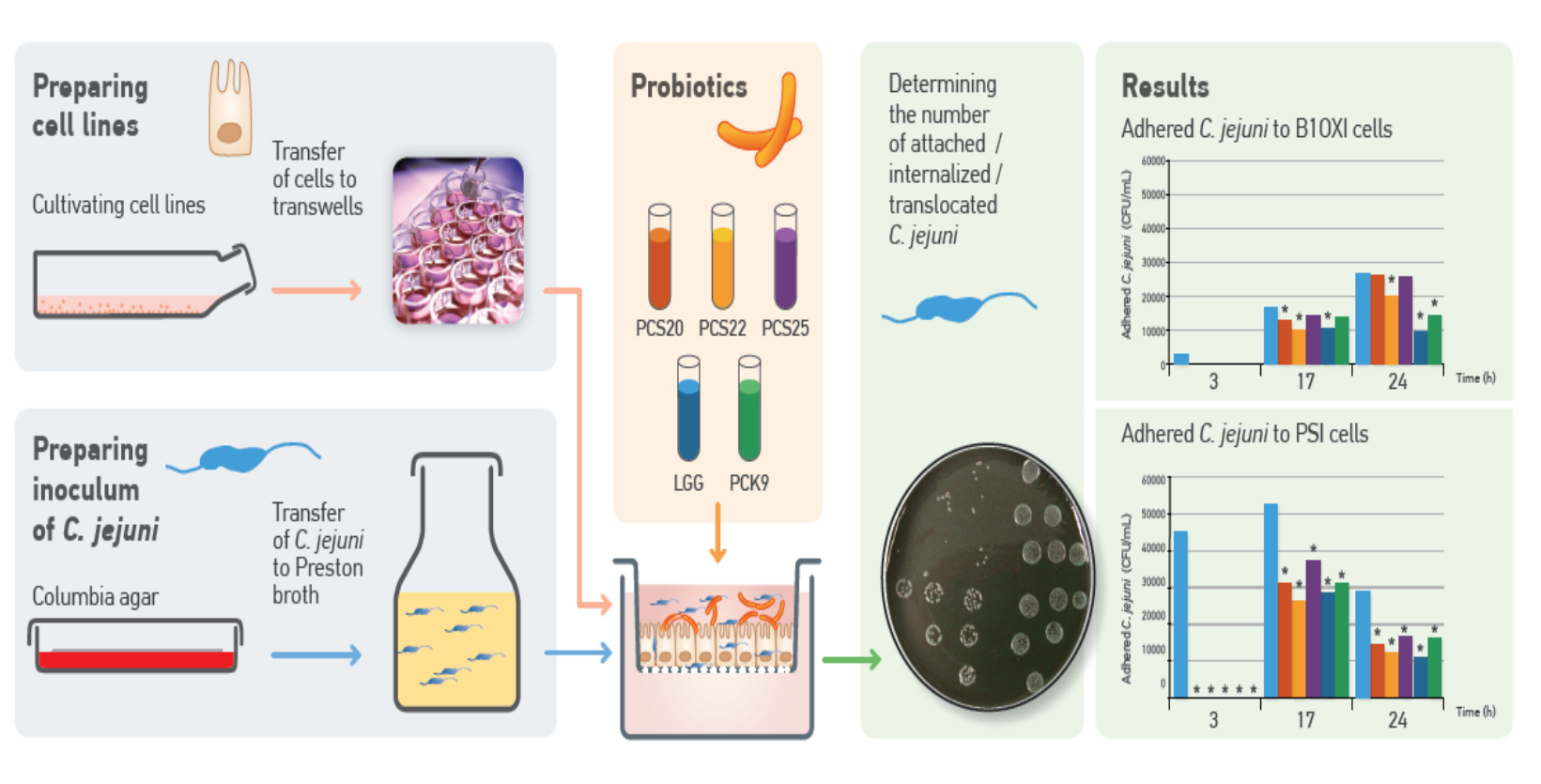

Supplement: Supplementary file 1 — Additional file 1. Visual scheme of experiment. We assessed the effect of different Lactobacillus strains on C. jejuni adhesion, invasion and translocation using pig (PSI) and chicken (B1OXI) enterocyte cell lines of non tumorigenic origin. [file 12917_2020_2238_MOESM1_ESM.docx]
